# Properties of Newly-Synthesized Cationic Semi-Interpenetrating Hydrogels Containing Either Hyaluronan or Chondroitin Sulfate in a Methacrylic Matrix

**DOI:** 10.3390/jfb3020225

**Published:** 2012-03-23

**Authors:** Annalisa La Gatta, Chiara Schiraldi, Antonella D’Agostino, Agata Papa, Mario De Rosa

**Affiliations:** Department of Experimental Medicine, Second University of Naples, Via L. De Crecchio 7, Naples 80138, Italy; Email: annalisa.lagatta@unina2.it (A.L.G.); antonella.dagostino@unina2.it (A.D.); agata_papa@virgilio.it (A.P.); mario.derosa@unina2.it (M.D.R.)

**Keywords:** pHEMA, hyaluronic acid, chondroitin sulfate, semi-IPNs, swelling, biocompatibility

## Abstract

Extracellular matrix components such as hyaluronan (HA) and chondroitin sulfate (CS) were combined with a synthetic matrix of p(HEMA-co-METAC) (poly(2-hydroxyethylmethacrylate-co-2-methacryloxyethyltrimethylammonium)) at 1% and 2% w/w concentration following a previously developed procedure. The resulting semi-interpenetrating hydrogels were able to extensively swell in water incrementing their dry weight up to 13 fold depending on the glycosamminoglycan content and nature. When swollen in physiological solution, materials water uptake significantly decreased, and the differences in swelling capability became negligible. In physiological conditions, HA was released from the materials up to 38%w/w while CS was found almost fully retained. Materials were not cytotoxic and a biological evaluation, performed using 3T3 fibroblasts and an original time lapse videomicroscopy station, revealed their appropriateness for cell adhesion and proliferation. Slight differences observed in the morphology of adherent cells suggested a better performance of CS containing hydrogels.

## 1. Introduction

A great number of naturally occurring polymers have been studied and proposed for the preparation of materials designed for Tissue Engineering applications [1].

Specific biological features, such as bioresorbability and cytocompatibility, coupled to novel biotechnological processes for manufacturing, are at the basis of growing biomedical interest in these macromolecules. On the other hand, natural polymers are soluble or rapidly degraded in the physiological environment, and also exhibit poor mechanical properties. In order to overcome these drawbacks, they are often proposed in combination with other polymers (both natural and synthetic) and/or in a chemically modified form (derivatized or crosslinked) [2,3,4,5,6,7,8,9,10]. 

In a previous work we reported the synthesis and the characterization of a multiphase system in which a biopolymer, alginate (sodium salt), was combined with a synthetic poly(2-hydroxyethylmethacrylate-co-2-methacryloxyethyltrimethylammonium) (poly(HEMA-co-METAC)) matrix in a semi-interpenetrating structure [11]. The resulting polyelectrolyte proved biocompatible and appropriate for cell adhesion and proliferation. In particular, the introduction of the biopolymer into the synthetic matrix considerably improved the biological response of the material. Additionally, the resulting hydrogel exhibited interesting swelling and mechanical properties, tunable with polysaccharide content [11]. Starting from this basis, the rationale of the present work was to synthesize similar semi-interpenetrating networks in which alginate was replaced by bioactive ECM components, such as hyaluronic acid (sodium salt) (HA) and chondroitin sulfate (sodium salt) (CS). In fact, hyaluronic acid, widely distributed throughout the ECM of all mammalian connective tissues, plays important roles in many fundamental biological processes, affecting several cellular functions, such as migration, adhesion and proliferation [2,3]. Chondroitin sulfate has been recently implemented in scaffold synthesis; it is the active principle of antiarthritic drugs [3,6,8,12,13,14,15] and recently, its biological functionality has been widely investigated, proving different effects in relation to the sulfation pattern [16].

From a tissue engineering perspective, the presence of ECM molecules in a scaffold is fundamental for creating an environment mimicking the cellular surrounding [3,6,7]. As a consequence, a better biological performance was expected for the novel combinations. Additionally, considering the differences in charge density of the polysaccharides, the inclusion of HA and CS in place of alginate in the positively charged p(HEMA-co-METAC) matrix should also regulate physical characteristics, particularly in terms of swelling behavior, reasonably widening the application field of such semisynthetic materials. 

Actually, both HA and CS have been proposed as the main components of scaffolds for tissue regeneration. Chang and co-workers (2003) have developed a gelatin-CS-HA tri-copolymer scaffold, also proving its potentialities in cartilage tissue regeneration [13]. Wang and co-workers (2007) developed a bi-layer gelatin/chondroitin-6-sulfate/hyaluronic acid membrane [3]. In their work, keratinocytes and dermal fibroblasts were found to attach and proliferate on the surface of the developed construct, suggesting the bi-layer membrane (with two different pore sizes) as a promising scaffold for a three-dimensional cell culture model in skin tissue engineering [3]. Type II collagen-CS-HA scaffolds, cross-linked by genipin, were also proposed for cartilage tissue engineering by Ko and collaborators in 2008 [12]. The introduction of CS in a type II-collagen scaffold increased chondrocyte proliferation and induced higher rates of GAGs synthesis, indicating possible stimulatory influence of CS on metabolic activity of seeded chondrocytes [6]. HA and CS have also been combined with synthetic polymers. For instance, poly(vinyl alcohol)-CS hydrogels, crosslinked by glutharaldehyde, were prepared and seeded with baby-hamster kidney cells, showing a sound biological response. In particular, the polysaccharide was found to promote cells-scaffold interaction, while the presence of PVA provided correct structure and architecture (suitable mechanical strength) [14]. Poly (lactide-co-glycolide) (PLGA)/HA blends were used to fabricate scaffolds by particle leaching and freeze-drying techniques. Cellular adhesion efficiency on the PLGA/HA blends was reported higher than on pure PLGA scaffold, probably because the addition of HA provided a most suitable contact angle for cell adhesion [17]. Besides improving cellular affinity, the increase in hydrophilicity due to the inclusion of HA was supposed to facilitate diffusion of nutrients inside the scaffold [17]. HA and CS have also been incorporated in methacrylic matrix. In particular, they were combined with neutral pHEMA matrix, producing materials negatively charged at physiological pH, which proved functional in different biomedical applications [18,19,20]. To the best of our knowledge, combinations of HA and CS with a synthetic positively charged (methacrylic) network have not yet been exploited. 

Bearing this literature background in mind, here we report the synthesis of semi-interpenetrating networks (semi-IPNs) in which HEMA and METAC were co-polymerized in the presence of aqueous solutions of HA or CS, and their characterization, in order to assess potential application in the biomedical field.

## 2. Materials and Methods

### 2.1. Materials

Commercial 2-hydroxyethylmethacrylate (HEMA) and 2-methacryloxy ethyltrimethyl ammonium chloride (METAC) were purchased from Sigma-Aldrich Chemicals Co. (St. Louis, MO, USA). HEMA is known to contain residual EGDMA and a stabilizer, hydroquinonone monomethyl ether (0.001%), due to the fabrication process. Monomers were used without further purification. Hyaluronic acid sodium salt lot N. 0901142 was purchased from BioPhyl (Milan, Italy), Chondroitin sulfate sodium salt, extracted from shark fin, was a kind gift from IBSA (Lugano, Switzerland). HA and CS disaccharide repeating units, along with structural formulas of HEMA and METAC monomers, are represented in Figure 1. 

α-α’ azoisobutyronitrile (AIBN) was purchased from Fluka (Milan, Italy). Dulbecco’s Phosphate Buffered Saline (PBS), Dulbecco’s Modified Eagles Medium (DMEM, high glucose, with glutamax^TM^), 3-(4,5dimethylthiazol-2-yl)-2,5-diphenyltetrazolium bromide (MTT), Fetal Bovine Serum (FBS), penicillin, streptomycin and fungizone were all purchased from Life Technologies (Breda, the Netherlands). Penicillin, streptomycin and fungizone were used at concentrations equal to 100 units/mL, 100 μg/mL, 2.5 μg/mL, respectively. 3T3 cells were purchased from ATCC (American Type Cell Collection, Manassas, VA, USA).

### 2.2. Biopolymers Hydrodynamic Characterization by SEC-TDA

The chromatographic analysis of the biopolymers was performed using the SEC-TDA (Size Exclusion Chromatography-Triple Detector Array) equipment by Viscotek (Lab Service Analytica, Italy). A detailed description of the system is reported elsewhere [21]. Two TSK-GEL GMPW_XL_ columns (Tosoh Bioscience, base material: hydroxylated polymethacrylate, pore size: 100–1000Å, mean particle size: 13 µm, dimensions: 7.8 × 30.0 cm, cat. N. 8-08025) in series that were preceded by a TSK-GEL guard column GMPW_XL _ (Tosoh Bioscience, mean particle size: 12µm, dimensions: 6.0 × 4.0 cm, cat. N. 08033) were used. An isocratic elution with 0.1M NaNO_3_ aqueous solution (pH 7) at a flow rate of 0.6 mL/min was carried out. Analyses were performed at 40 °C with a running time of 1hr. Sample molecular weight (M_w_, M_n_, M_w_/M_n_), molecular size (hydrodynamic radius-R_h_) and intrinsic viscosity ([η]) distributions were derived. The MHS curves (log [η] *vs.* log M_w_) and, thus, the related constants were also directly obtained [21].

### 2.3. Hydrogel Synthesis

The hydrogels were synthesized following a previously described procedure [11]. Briefly, HEMA and METAC monomers were co-polymerized in the presence of aqueous solutions of each GAG (HA or CS) at 1% and 2% w/v final concentrations using AIBN as thermal initiator. In particular, aqueous solutions of each biopolymer (2% and 4% w/v) were added under stirring to HEMA/METAC mixtures (10:1 w/w) in 50/50 volume ratio and AIBN (0.1% w/w with respect to HEMA+METAC weight) was finally added. A control mixture was prepared using water in place of GAG solutions. Each mixture was poured between two glass plates overlapped with two 3M transparency films (3M Visual Systems Products, Europe, France) spaced by a silicon rubber (thickness of 1 mm) to obtain uniform hydrogels membranes. The samples were cured at 60 °C for 1 hr, 70 °C for 16 hr and 85 °C for 1hr in a forced-air circulation oven. After curing, the resulting materials, to which we will refer as p(HEMA-co-METAC)/H_2_O, p(HEMA-co-METAC)/HA1%, p(HEMA-co-METAC)/HA2%, p(HEMA-co-METAC)/CS1% and p(HEMA-co-METAC)/CS2%, were removed from the 3M transparency films and washed three times in de-ionized water for 24 hr to remove residual unreacted monomers. The rectangular polymeric membranes, in the swollen state, were cut to a circular shape to fit into a 12-well plate for chemico-physical and biological characterization and then dried in a forced-air circulation oven at 40 °C for 48–72 h. 

### 2.4. Swelling Studies

The water uptake for each material was studied in de-ionized water and in physiological solution (0.15 M NaCl, 150 mOsm/L). The swelling kinetic and the equilibrium swelling of the hydrogels were evaluated. 

In all cases, the water uptake was determined by gravimetric measurements using an analytical balance (Mettler Toledo, XS105 Dual Range). In particular, materials were immersed into the swelling solutions (200 mL aqueous medium/g of sample) and kept in a thermostatic bath at 37 °C. Specimens were removed at fixed intervals, 15–30 min up to 5 hr for kinetic studies, or after 24hr to assess the equilibrium swelling degree (equilibrium studies). Withdrawn samples were then blotted with filter paper to remove surface water and finally weighed. The swelling degree and the swelling ratio were calculated as follows:

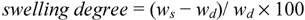
(1)

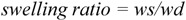
(2)
where *w_s_* = swollen sample weight; *w_d_* = dried initial sample weight. Experiments were run in triplicate.

### 2.5. Biopolymer Release Studies

Biopolymer release from the polyelectrolyte matrices was determined through the following procedure: specimens of the dried materials (about 500 mg) were immersed in physiological solution (40 mL/g) and kept under stirring (200 rpm) at 37 °C for one week. At increasing time intervals, 1.5 mL of the medium were withdrawn and analyzed for the biopolymer content through the carbazole assay [22]. The amount of released biopolymer was calculated as:

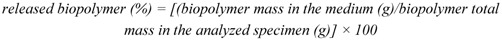
(3)

### 2.6. Cell Culture

3T3 fibroblasts were routinely cultured in DMEM, supplemented with FBS (10% v/v), nonessential amino acids (1% v/v) and antibiotics. Cells were maintained at 37 °C in a 5% CO_2_, 95% air, humidified atmosphere and media were changed every 48 h.

### 2.7. *In Vitro* Cytotoxicity Tests

The developed materials were tested for *in vitro* cytotoxicity to assess the suitability of their use in biomedicine. The *in vitro* cytotoxicity was evaluated by means of the elution test method (ISO 10993-5), exposing 3T3 fibroblasts grown to near confluence to fluid extracts from the materials under investigation as accurately described elsewhere [23], and then evaluating cell viability, after 48 hr of incubation, by MTT assay [24]. Cell viability was calculated as percentage with respect to the control (cells incubated in fresh culture medium) [23].

### 2.8. Cell Adhesion and Proliferation

Cytocompatibility was evaluated for all the materials obtained using 3T3 fibroblasts. In particular, p(HEMA-co-METAC)/H_2_O, p(HEMA-co-METAC)/CS1%, p(HEMA-co-METAC)/CS2%, p(HEMA-co-METAC)/HA1% and p(HEMA-co-METAC)/HA2% were treated twice with PBS containing antibiotics and then equilibrated overnight in DMEM at 37 °C in a 5% CO_2_ humidified atmosphere. Cells were then seeded on each disk (1–2 × 10^4^ cells per cm^2^) and placed in a stage incubator of a time lapse videomicroscopy station in order to continuously monitor cell behavior in a 72 h experiments.

The time lapse station (OkoLab, Naples, Italy) is composed by a inverted optical microscope (Axiovert 200; Zeiss, Oberkochen, Germany), a motorized stage incubator (air/CO_2_), a thermostatic bath (Lauda, Eco Line RE204, Frankfurt, Germany) and a motor control. The station is equipped with an innovative software (OKO-Vision 2.7, Naples, Italy) that permits a continuous on-line following of the experiment and the performing of images analysis. In particular, at about 3 hr after seeding, different areas on the hydrogel surfaces seeded with cells were selected. The software automatically drove the stage to return with micrometric precision on each selected X, Y, Z position at established time intervals, acquiring an image through a CCD gray-scale camera (Hamamatsu, Germany). Images relative to each area were automatically stored. Overall, the system was allowed to follow the movement of selected cells and, eventually, their adherence, spreading and proliferation on the material surface. The software was also used for image analysis in order to evaluate cell proliferation on each material. In particular, the adherent cells/cm^2^ were counted at time zero and every 12 h in 72 h experiments (proliferation curves were obtained by plotting cells number/cm^2^ *vs.* time). 

The possibility to contemporaneously incubate more samples and also to visualize within the same sample several fields of view ensures the statistical significance of the experiment. 

## 3. Results

### 3.1. Biopolymer SEC-TDA Characterization

HA and CS were analyzed by SEC-TDA to accomplish a complete hydrodynamic characterization. The report of the SEC-TDA analyses is presented in Table 1. HA was a high molecular weight polysaccharide with a M_w_ of 1300 ± 40kDa, a polydispersity index of 1.4 ± 0.1. CS was a low molecular weight polysaccharide with a M_w_ = 36 ± 1kDa, a polydispersity index of 1.4 ± 0.1. As expected, the intrinsic viscosity of the longer chain biopolymer was about 20-fold the one of CS. The MHS constants (“a” and “log k”) for each biopolymer are also reported in the Table 1. (Please use the MS table function to redraw table 1.)

**Table 1:** Values of weight average molar mass (M_w_), numeric average molar mass (M_n_), polydispersity index (M_w_/M_n_), intrinsic viscosity ([η]), hydrodynamic radius (R_h_) and Mark-Houwink constant “a” and “logk” for the biopolymers used in the synthesis of the semi-IPNs hydrogels as obtained by SEC-TDA analyses.

Biopolymer	M_w_ (kDa)	M_n_ (kDa)	M_w_/M_n_	[η] (dL/g)	R_h_ (nm)	a	log k
HA	1300 ± 40	980 ± 20	1.4 ± 0.1	21 ± 1	70 ± 1	0.60 ± 0.01	−2.75 ± 0.05
CS	36 ± 1	25 ± 1	1.4 ± 0.1	0.9 ± 0.1	7.8 ± 0.1	0.96 ± 0.01	−4.46 ± 0.05

### 3.2. Swelling Studies

**Figure 2:**
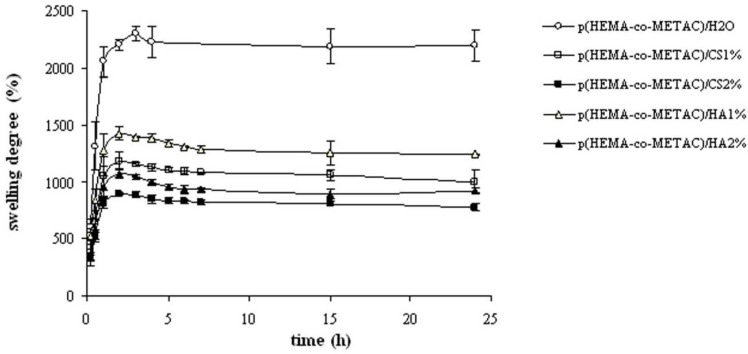
Swelling curves in deionized water at 37 °C for the novel hydrogels and for p(HEMA-co-METAC)/H_2_O.

**Figure 3:**
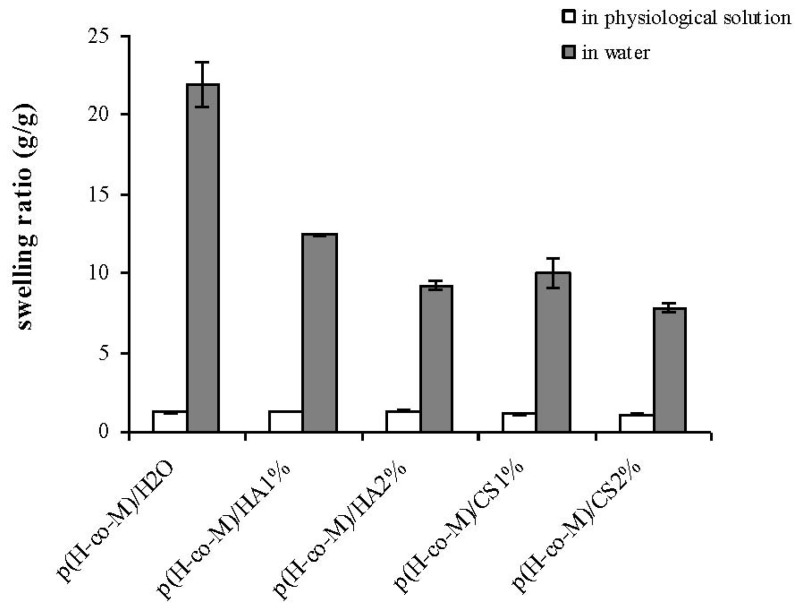
Swelling ratio values (hydrated material weight/dried material weight) for the novel hydrogels and p(HEMA-co-METAC)/H_2_O as measured at 37 °C in physiological solution (0.15M NaCl, 150mOsm/L). Swelling ratio values in water are also reported for an immediate comparison. p(H-co-M), as reported in the figure, stands for p(HEMA-co-METAC).

Figure 2 shows the water swelling kinetics for the novel hydrogels compared to the one of p(HEMA-co-METAC)/H_2_O. The samples evidenced rapid swelling and reached equilibrium within 4 hr. All hydrogels, as charged networks, presented high values of swelling degree (> 100%). As expected, the latter for the semi-interpenetrating hydrogels was considerably lower than that of the p(HEMA-co-METAC)/H_2_O, and decreased with the increase of the biopolymer content. Further, it is evident that, despite the same biopolymer concentration, the hydrogels containing CS showed a lower water uptake capacity with respect to the ones containing HA. In Figure 3, the equilibrium swelling ratio in physiological solution is reported together with the respective values in water for an immediate comparison. As expected, at 0,15M NaCl, all the charged hydrogels showed considerably lower water uptake compared to the one in deionized water. The differences in the swelling behavior of hydrogels became negligible when swollen in physiological solution. 

### 3.3. GAGs Release from the Semi-IPN Hydrogels

**Figure 4:**
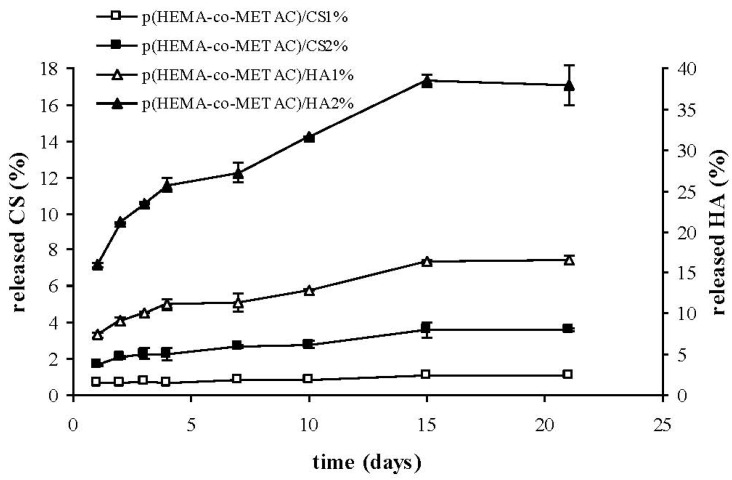
Amount of released polysaccharide (percentage with respect to the total amount of the biopolymer in the hydrogel) from the semi-IPNs during incubation in physiological solution at 37 °C.

Results of the release studies are reported in Figure 4. The amount of HA released in 21 days experiments from p(HEMA-co-METAC)/HA2% and p(HEMA-co-METAC)/HA1% was about 38% and 16%, respectively. The semi-IPNs containing CS released less than 5% of the initial polysaccharide. Biopolymer release was superior for the 2% GAG containing hydrogels, and the highest releasing rate was found in the first day’s incubation followed by a *plateau*. 

### 3.4. *In Vitro* Cytotoxicity Tests

The developed hydrogels were found not to be cytotoxic: viability of 3T3 fibroblasts (evaluated as described in Materials and Methods section) after 48hr of incubation in culture media conditioned with the tested hydrogels was at least 80% with respect to the control (data not shown). 

### 3.5. Cytocompatibility

**Figure 5:**
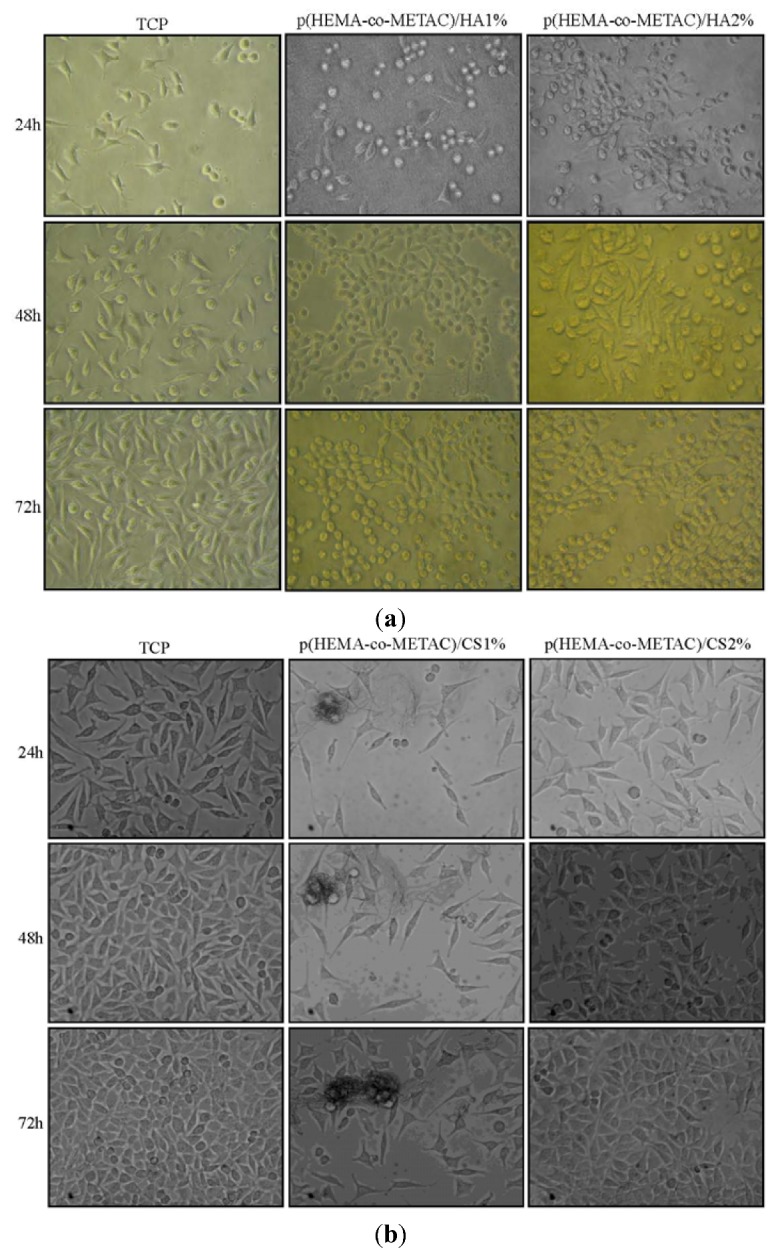
Panel of micrographs of 3T3 murine fibroblasts at 24, 48 and 72hr incubation in the time lapse station on (**a**) TCP (control), p(HEMA-co-METAC)/HA1% and p(HEMA-co-METAC)/HA2%; (**b**) TCP (control), p(HEMA-co-METAC)/CS1% and p(HEMA-co-METAC)/CS2%.

Micrographs of 3T3 fibroblasts on materials at 24, 48 and 72 h after seeding and the relative control (cells on TCP), as acquired by the time lapse station, are shown in Figure 5 (panels a and b). The panel in Figure 5b shows sequences of micrographs of the same area during incubation (precise repositioning of the stage-incubator), thus allowing proliferation to be followed. In particular, it can be observed that cells proliferated on p(HEMA-co-METAC)/CS2%, similarly to TCP, while proliferation on p(HEMA-co-METAC)/CS1% was lower. Morphology of adherent cells was consistent with the cell type on both the CS containing materials. In the case of HA containing materials (panel a, Figure 5), observation was more difficult, due to the higher opacity of the semi-IPN. However, many of the acquired pictures established that HA semi-IPN was comparable to the control on the spreading and only few adherent cells presented a round-shaped morphology instead of an elongated one. 

**Figure 6:**
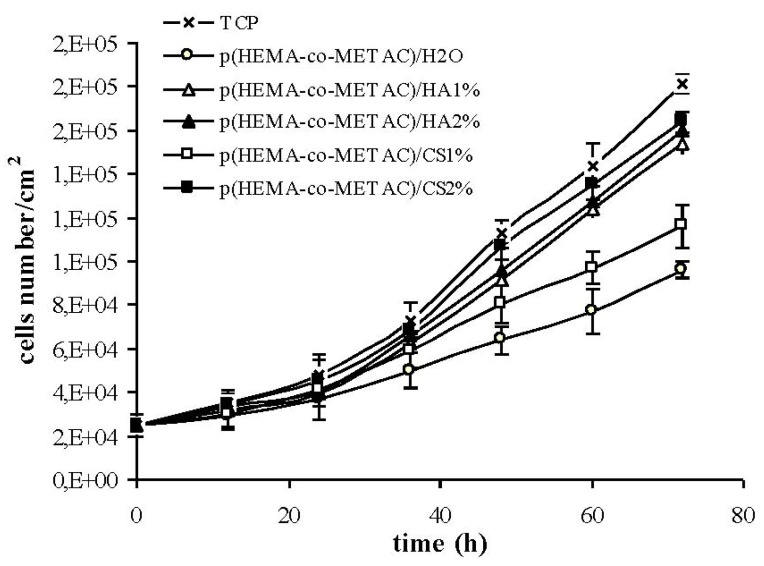
3T3 murine fibroblast proliferation on hydrogel surfaces and on TCP (control).

Proliferation curves (cells number/cm^2^ *vs.* incubation time, normalized with respect to the number of seeded cells) for the novel materials compared to the control and to p(HEMA-co-METAC)/H_2_O are reported in Figure 6. As shown, results for p(HEMA-co-METAC)/CS2% and both the HA containing materials were the most similar to the control (proliferation-rate 0.025 h^−1^ *vs.* 0.027 h^−1^ of the control) while p(HEMA-co-METAC)/CS1% showed a minor growth-rate (proliferation-rate 0.022 h^−1^). The novel materials sustained proliferation better than the copolymer synthesized in the presence of water (growth-rate 0.022 h^−1^).

## 4. Discussion

Considering that the combination of sodium alginate with a synthetic, positively charged, p(HEMA-co-METAC) matrix in a semi-interpenetrating structure gave materials (with improved physical and biological features with respect to the sole synthetic matrix) novel combinations have been exploited in the present work. In particular, p(HEMA-co-METAC) was combined with GAGs: specifically, HA and CS. Given that such polysaccharides are ECM components, the resulting materials were expected to be particularly suitable in mimicking cellular environment and predictably functional in specific applications [3,5,6,7,8]. It was also expected that the introduction of different polysaccharides into the matrix would determine different physical properties, especially in terms of swelling behavior, reasonably tuning final material properties dependent on the polysaccharide nature and content. 

Swelling properties of the novel hydrogels were investigated in water and in physiological solution (Figure 2 and Figure 3) since these proved important in view of application (e.g., nutrient transport, drug delivery). The swelling kinetics in water (Figure 2), as expected, demonstrated the high water uptake capacity of the developed materials with swelling degree values in the range 800–1,400%. As shown, water uptake decreased with the increase in the GAGs content. This is reasonably due to the partial neutralization of the positive charges of the co-polymer by the carboxylate (and sulfate) groups of the polysaccharides. The resulting minor net charge of the matrix is responsible for a decrease in chain relaxation and hydration and in ion osmotic swelling pressure, thus lessening the water uptake capacity [11]. Further, the co-polymerization in the presence of aqueous solutions of the biopolymers in place of water is expected to result in more dense materials, thus contributing to diminished swelling. Additionally, it is evident that the materials containing CS exhibited a lower water uptake compared to the ones containing HA at each GAG concentration. In fact, considering the sulfation pattern of shark fin derived CS [25], the number of negative charges introduced using CS is 1.64 fold the ones introduced using HA at the same weight percentage. It is interesting to note that the swelling degree in water for the materials containing HA and CS was higher with respect to the values found for the same matrices containing alginate, thus suggesting the possibilities of diverse applications [11]. In fact, at the same weight percentage, the alginate presents a higher number of negatively charged groups (1.22 fold with respect to CS and 2.02 fold with respect to HA) that counterbalance the NR_4_^+^ moieties.

As expected, the water uptake of the hydrogels decreased when swollen in physiological solution due to the minor ion osmotic pressure (the difference in concentration of mobile ions between the gel and the solution is reduced when increasing the ionic strength of the swelling medium), and no significant differences among the diverse materials were recorded. This last result confirms our previous findings on p(HEMA-co-METAC)/alginate hydrogels [11], indicating ion osmotic pressure as the primary driving force in the swelling of the semi-IPNs in water. 

As shown in Figure 4, the developed semi-IPNs released the polysaccharide to a certain extent. In particular, the percentage of biopolymer released with respect to the total amount present in the materials was considerably higher for HA than for CS, reasonably due to the stronger ionic interactions established between CS and the positively-charged synthetic matrix. Despite the fact that such interactions are known to become stronger with increased polymer molecular weight, our results proved that the higher molecular weight HA (1.3 MDa) released more than CS (36 kDa). This indicates a major effect of the charge density on the interaction between GAGs and the cationic matrix. This last finding is in agreement with data reported in literature regarding chitosan; in fact it was shown that the structural densities of chitosan/chitosan sulfate complexes were strongly affected by polymer charge density (acetylation degree) rather than molecular weight [26]. The partial release of hyaluronic acid from hydrogels on contact with the biological environment (Figure 4) could turn into an advantageous aspect, since HA is expected to interact with cell specific receptors, eventually promoting cellular migration, and thus wound healing, as reported in literature [27,28,29].

The absence of harmful extractables from the synthesized hydrogels was verified by indirect cytotoxicity tests performed according to ISO 10993-5 (data not shown). Further, in an attempt to propose synthesized materials for tissue engineering and to biologically compare them to the ones containing alginate, proliferation and morphology of cells on their surfaces have been investigated. As shown in Figure 5 and Figure 6, the developed hydrogel surfaces produced a cytocompatible result. In particular, the introduction of GAGs in the synthetic matrix clearly enhanced cellular proliferation (Figure 6) with p(HEMA-co-METAC)/CS 2% semi-IPN showing the most promising results (Figure 5 and Figure 6). 

It is worth mentioning that although HA and CS, as ECM components, are recognized as ideal scaffold materials, controversial literature exists on the effect of the polymers on cell adhesion. In particular, the polyanionic nature of HA was reported to thermodynamically not favor cellular adhesion [1,30]. Similar observations were described for heparin-based materials which showed anti-adhesive properties with respect to human aortic adventitial fibroblasts; the authors suggested that either the bulk negative charge or the specific interactions established between heparin and cells could be at the basis of such behavior [31]. As mentioned, HA and CS were already incorporated in a neutral pHEMA network, producing materials which proved functional (a) as drug release systems; (b) as new contact lens materials with reduced protein adsorption, and as (c) as materials to prevent adhesion following surgery [18,19,20]. The latter are certainly biocompatible materials, however, cell recognition and interaction, at the basis of proliferation and thus tissue regeneration, is not found/reported. This behavior could be related to the net negative charge of the final networks. Contrariwise, the net charge of the semi-IPNs presented remains positive, despite the polyanionic nature of HA and CS, thus reasonably favoring cell attachment [30]. 

Overall, the combination of GAGs with a positively-charged synthetic matrix should allow an adhesive material to be obtained, due to the cationic surface, a mimicking cellular environment due to the GAG content and to contemporaneously tune physical properties by varying the nature and the content of the polysaccharide.

Finally, it is worth underlying that, as expected, when using hyaluronan and chondroitin sulfate, the overall cellular response to the semi-IPNs improved with respect to the one found for p(HEMA-co-METAC)/alginate hydrogels [11]. 

## 5. Conclusions

HA and CS were successfully introduced into p(HEMA-co-METAC) matrices, producing GAGs-based semi-interpenetrating hydrogels. The swelling properties of the developed materials were consistent with their polyelectrolyte nature and with the content and charge density of the biopolymer included. The outstanding water uptake and the partial release of the GAGs that was observed upon contact with aqueous medium potentially have advantageous effects in biomedical applications with respect to the previously synthesized p(HEMA-co-METAC)/alginate hydrogels. All developed materials proved biocompatible and suitable for cell adhesion and proliferation.

## Supplementary Files

Supplementary File 1 DOC-Document (DOC, 29 KB)

Supplementary File 2 PNG-Document (PNG, 40 KB)
