# Supplementary material for: Properties of Newly-Synthesized Cationic Semi-Interpenetrating Hydrogels Containing Either Hyaluronan or Chondroitin Sulfate in a Methacrylic Matrix
Source: J Funct Biomater. 2012 Mar 23;3(2):225–38. doi: 10.3390/jfb3020225 (PMC4047938; doi:10.3390/jfb3020225)
Supplement: Supplementary File 1 — DOC-Document (DOC, 29 KB) [file jfb-03-00225-s001.doc]

| **Biopolymer** | **M_w_ (kDa)** | **M_n_ (kDa)** | **M_w_/M_n_** | **[η] (dL/g)** | **R_h_ (nm)** | **a** | **log k** |
| --- | --- | --- | --- | --- | --- | --- | --- |
| HA | 1300 ± 40 | 980 ± 20 | 1.4 ± 0.1 | 21± 1 | 70 ± 1 | 0.60 ± 0.01 | -2.75 ± 0.05 |
| CS | 36 ± 1 | 25 ± 1 | 1.4 ± 0.1 | 0.9 ± 0.1 | 7.8 ± 0.1 | 0.96± 0.01 | -4.46± 0.05 |

**Table 1** Values of weight average molar mass (M_w_), numeric average molar mass (M_n_), polydispersity index (M_w_/M_n_), intrinsic viscosity ([η]), hydrodynamic radius (R_h_) and Mark-Houwink constants “a” and “logk” for the biopolymers used in the synthesis of the semi-IPNs hydrogels as obtained by SEC-TDA analyses.
