# Supplementary figures and images for: Properties of Newly-Synthesized Cationic Semi-Interpenetrating Hydrogels Containing Either Hyaluronan or Chondroitin Sulfate in a Methacrylic Matrix
Source: J Funct Biomater. 2012 Mar 23;3(2):225–38. doi: 10.3390/jfb3020225 (PMC4047938; doi:10.3390/jfb3020225)

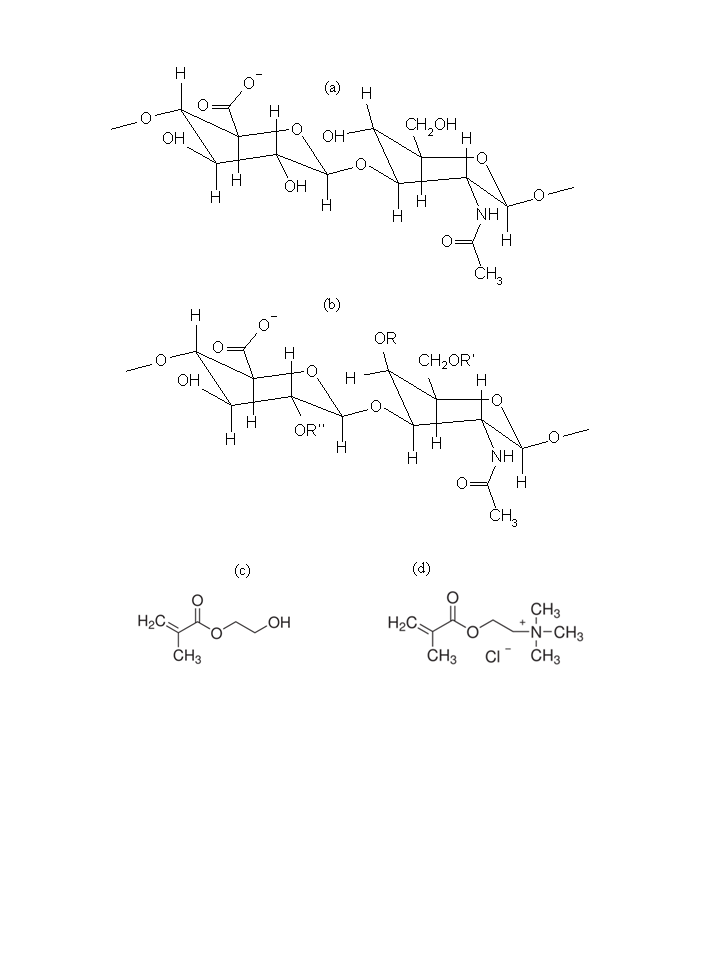

Supplement: Supplementary File 2 — PNG-Document (PNG, 40 KB) [file jfb-03-00225-s002.png]
